# Simulation as an effective means of preparing trainees for active participation in MDT meetings

**DOI:** 10.1016/j.fhj.2024.100017

**Published:** 2024-02-28

**Authors:** Ewan Christopher Mackay, Kishen Rajan Patel, Colette Davidson, Jessica Little, Karen Tipples, Adam Januszewski, William Ricketts

**Affiliations:** a King's College Hospital NHS Trust, Denmark Hill, London, SE5 9RS, UK; b Barts Health NHS Trust, London, London, UK; c University College London, London, UK

**Keywords:** Simulation, MDT, Cancer, Training

## Abstract

**Introduction:**

Cancer multi-disciplinary team (MDT) meetings are an important component of consultant workload, however previous literature has suggested trainees are not satisfied with their current curriculum in preparing for MDT working.

**Methods:**

This educational pilot assessed whether multi-speciality simulated scenarios with pre-defined learning objectives, could prepare specialist registrars for interacting within an MDT. Participants completed pre- and post-questionnaires assessing a number of areas including: current experience of training, confidence presenting patients and whether the course would alter future practice.

**Results:**

Trainee confidence increased significantly from a mean of 5 to 7 (mean to nearest whole number, *p* < 0.01). Trainees rated the session highly for utility and altering their future practice (mean scores of 9 for both respectively, out of 10).

**Conclusion:**

Simulation has shown success in other multidisciplinary teaching, however to our knowledge there are no cancer specific training programmes. Our results highlight a potential gap in UK specialist training, and suggest simulation may be beneficial in preparing trainees to present in MDT meetings.

## Introduction

Multi-disciplinary team (MDT) meetings are a staple part of work for speciality doctors in the UK and have been shown to improve patient care.^1^ Treatment of cancers is dependent on a variety of different factors, including the type, position and stage of cancer, as well as the person's underlying co-morbidities and fitness.^2^ Given the increasing complexity of modern medicine, having viewpoints from multiple specialities ensures that patients are managed holistically according to best practice.^3^ This is especially important for complex cases with multiple coexisting comorbidities, which are likely to rise with an ageing population and is increasingly being seen in many western countries.^2^

Simulation training has been used by medical educators to teach a variety of different clinical skills including management of emergencies, cardiopulmonary resuscitation and procedural skills.^4^ It has also demonstrated utility in integrating members of multi-disciplinary teams who normally work together.^5^ Previous literature to our knowledge has not however looked at using simulation as a means of teaching the skills required in a cancer MDT to present and manage complex patients.

Lung cancer is the third most common cancer in the United Kingdom^6^ and has a significant burden on the health service with over 40,000 people diagnosed with lung cancer in 2015.^6^ Lung cancer MDT meetings are complex with a number of different members including: Respiratory Physicians, Medical Oncologists, Clinical Oncologists, Thoracic Surgeons, Radiologists, Pathologists and Clinical Nurse Specialists to name but a few. However, 50% of Respiratory and Oncology trainees in London had previously reported they were dissatisfied with their training experience in the cancer MDT and were not achieving their curriculum objectives. Trainees reported that they were not having the opportunity to present cases, have meaningful input in clinical decision making and found the educational benefit within the MDT setting limited.^7^

MDT meetings make up a substantial proportion of Respiratory and Oncology consultant workload, but despite their importance in patient care, trainees have struggled to meet curriculum objectives and this may have been exacerbated during the covid pandemic with virtual interfaces replacing many in-person meetings.^8^

The primary aim of this educational project was therefore to assess whether a simulated cancer MDT (integrating trainees from different specialities) could be an effective means of improving trainee confidence discussing patients in MDT meetings, lead to a change in their practice, and prepare specialist doctors for the leadership roles they will likely require as a Consultant.

## Methods

Respiratory and Oncology trainees undergoing Higher Specialty (Registrar) Training across London were invited to attend a 3 h interactive MDT simulation session. The programme was mapped closely to Capabilities in Practice (CiPs) found in the relevant curricula from the Royal College of Physicians (RCP)^9^ and Royal College of Radiologists^10^ to ensure relevance to training requirements and quality control. The majority (16) of the attendees were in person, with three joining virtually.

MDT scenarios were designed to cover a variety of commonly encountered situations varying from those focussed more on non-technical skills (human factors) to more complex clinical scenarios. Five scenarios were written by education fellows with review by subspeciality cancer leads. Each scenario had pre-defined learning objectives and a summary is included in Table 1. These were designed to mirror the complexities seen in real life and to prepare candidates for interacting with multiple specialities, challenging colleagues if necessary, whilst maintaining professionalism and relationships within the meeting structure. Four scenarios were included in each session.

**Table 1:** A table to demonstrate examples of scenarios presented at simulated MDT meeting.

Scenario	Focus of
1	Dealing with difficult/disengaged colleagues to promote best patient care.
2	Patient likely to require systemic treatment but limited initial clinical information requiring interaction with multiple members of MDT.
3	Not enough clinical information available requiring careful navigation of team members who are emotionally involved in the case.
4	Cancer recurrence in patient with borderline fitness for further diagnostic tests. Presented by oncology team.
5	Patient presented at diagnostic MDT that although not a candidate for curative/ systemic therapy has a high acute symptomatic burden requiring consideration of appropriate symptomatic treatment/palliative care.

For each scenario, a Respiratory and Oncology trainee played the role of a newly qualified Consultant in their field, leading the presentation and discussion of fictional patients, interacting with each other as well as various other specialist teams as required. Other specialities including: Radiology, Surgery, Histopathology and Nursing staff had scripted roles which were played by faculty staff. Trainees did not take roles outside their speciality to maintain fidelity.

Each simulation typically lasted between 10 and 15 min to reach its natural conclusion and was followed by a focussed debriefing session by both Subspeciality Consultant leads (with an education background) as well as Medical Education Fellows. The DAA (Describe, Analyse, Apply) or Diamond model is a validated simple tool that was used to structure debriefing after scenarios, which first focusses on describing events, then in turn analysing their effectiveness, before finishing with discussing the application of learning events to future practice.^11^

Trainees completed a pre- and post-course questionnaire, which assessed perceptions of their current training programme, confidence presenting patients, the utility of the session and if it would alter their clinical practice moving forwards. Visual analogue scales were used to score questions from 1 to 10 depending upon their relevance and paired *t*-tests were used to assess statistical significance. Free text answers for some questions were also included to allow further appraisal and development of the programme.

## Results

Nineteen trainees attended a 3-h simulation session, with participation in four simulated scenarios from a bank of cases written by the authors. All grades of registrar were included from ST3 to ST7, of which eight were senior trainees (ST6+). All participants were included in the analysis. This included eleven Oncology and eight Respiratory trainees, with the mean number of MDTs attended by participants in their current training posts at 1.3 per week. As previously mentioned, the teaching session was hybrid; 16 participants attended in person, and three joined virtually via Microsoft Teams (Version 1.5, Microsoft Corporation, Washington).

**Fig. 1:**
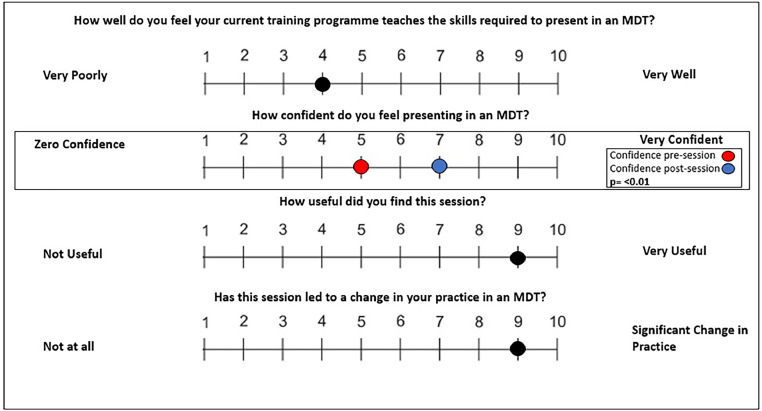
A visual analogue scale to display trainee responses to questions regarding MDT meetings. *n* = 19.

When asked how well their current training programme prepared them for the skills required to present in an MDT on a 10-point Likert scale (with 1 being rated as very poor and 10 as very well), trainees rated their current programme 4 (all means rounded to nearest whole number; range 2–10, see Fig. 1).

The confidence of trainees who participated in the simulation programme significantly increased from 5 at baseline (mean; range 3–7) to 7 (mean, range 6–9; *p* < 0.01) after the session.

Trainees rated the session's overall usefulness at 9 (mean, scale 1–10; range 7–10). When asked how likely the session would lead to a change in practice, (from 1 – no change at all, to 10 – significant change in practice) the trainee response was 9 (mean; range 6–10).

When asked how this would lead to a change in their practice moving forwards, trainees in free text commented on aspects such as: ‘signposting the relevant bits, handling disengaged colleagues, ensuring we include all members of the MDT in discussion', ‘use your colleague's expertise' and ‘understanding the roles and limitation of the MDT'. When asking what aspects of the session were particularly useful for their learning, four of the 19 responses commented on the involvement of both Respiratory and Oncology trainees, as well as debriefs being led by consultants in both specialities. This was displayed in comments such as: ‘Very useful to have resp[iratory] colleagues there. We very rarely have this kind of interaction with fellow specialities' and ‘Good to have Respiratory and Oncology consultants on site as good to get Oncology perspective on cases.'

## Discussion

Simulation training has previously shown success in improving confidence within the wider concept of a multi-disciplinary team.^5^ Literature looking at improving MDT outcomes has focussed mainly on seminars and debrief sessions following meetings, with limited application to oncology meetings.^12^ To our knowledge, a study has not previously looked at improving presentation skills in cancer MDTs using inter-disciplinary simulated roles and our work is novel in this area.

### Analysis of results

These results add to the growing body of literature^7^ which suggests current UK Specialty Training programmes do not provide adequate preparation for presentation and discussion in MDT meetings, with a trainee rated mean score of 4 (from 1 to 10) when asked how well they felt their current training programme taught them the skills required to present in an MDT meeting. Given the importance of cancer MDT meetings in optimising patient care and promoting holistic care, this perceived lack of training should not be ignored.^3^

Trainees rated the session very highly in terms of utility and likelihood of altering their future practice (mean scores of 9 out of 10 respectively for both) and trainee confidence post intervention demonstrated a statistically significant increase of 2 (from 5 to 7, *p* < 0.01). This suggests that simulation may well have a role in helping to provide training and is likely under-utilised in this regard.

By using simulation training, our aim was to focus on experiential learning, a style of learning originally described by David Kolb who has suggested a framework for ‘learning from life experience’.^13^ Our scenarios were designed with this framework in mind; trainees actively participated in presenting patients (creating a concrete experience for their own reference), then an active debriefing session stimulated reflection and abstract thinking. The debrief and post intervention questionnaires focussed on how the session may change the future practice of trainees, to encourage active experimentation in future situations.

Our approach challenges the traditional medical model of postgraduate learning, which is often described as an apprenticeship. Many authors such as Lave and Wenger,^14^ have long contested that learners develop from legitimate peripheral participation, into more active participants on their route to becoming ‘masters’ themselves.^14^^,^^15^ Often MDT meetings are busy, with a high caseload, and decisions have to be made quickly without reasoning being explained to junior members of the team.^12^ Our results would suggest that, in this context, simply attending busy meetings does not support learning in the apprenticeship manner described by Lave and Wenger^14^ as the trainee's participation is too passive to impart educational value or enable confidence building in skills such as presentation. The purpose of this simulated MDT was therefore to form a bridge that allowed trainees to transform their role from passive observers to active members of the MDT meeting. With a significant increase in confidence demonstrated, the sessions appeared to be successful in this regard.

### Limitations and future research

Although our project demonstrated promising results in this area, it was not without its limitations and there are areas to build on with future projects. Although our data demonstrated an improvement in confidence and a suggestion of impact on future practice, it is not clear whether this will be directly carried over into clinical practice or be sustained for the longer term. A number of interventions might be used to build on this further.

This project primarily looked at trainee confidence in MDT meetings which is likely very important to trainees, but does not necessarily equate to increased competence in the presentation and management of patients in an MDT environment. Measurable metrics such as expert assessment that could be performed both pre- and post-session, might represent a more objective and robust method of assessing if these sessions have indeed improved trainee performance. In addition, formative assessment methods can be successful as an educational tool and this approach could potentially add to the educational value of the sessions themselves.^16^

Considering re-assessment at a set interval such as 6 or 12 months would also add a valuable insight into if there is a maintenance of change and how this effect varies over time. This future approach has many advantages but may run the risk of potentially deterring trainees from attending, and psychological safety would have to be managed if trainees feel that they may be assessed throughout the process.

It is also important to consider the potential bias in the trainees that were included in the study. The sessions were advertised widely to speciality trainees across London, but it could be argued that trainees who would sign up for such a simulation session to improve their MDT presentation skills, may be more receptive to learning in such a format. Although it is hoped that the debriefing sessions are dynamic enough to engage different types of learner; it cannot be assumed that the results are necessarily generalisable to all trainees and learning styles. However, this bias is difficult to overcome within a voluntary teaching programme.

To conclude, this educational pilot was conducted to assess the effectiveness of simulated scenarios as a training tool for conducting MDT meetings. The scenarios were designed to develop confidence in presenting patients and promote inter-disciplinary working. Trainees reported a significant increase in their confidence levels and found the session useful in preparing them for future clinical practice. Overall, this suggests that simulated scenarios can be an effective tool for preparing speciality doctors for the roles required in an MDT setting. Following the success of this programme, further research is warranted to investigate if the lung cancer simulated MDT model used here could serve as a paradigm for other MDT meetings which, in the first instance, might involve expanding to other specialities (e.g. Radiology, Pathology and Surgical sub-specialities) or tumour sites.

## Declaration of competing interest

There are no competing interests for any of the authors.
